# Dental Manipulation

**Published:** 1876-01

**Authors:** A. Doud

**Affiliations:** Olathe, Kan.


### DENTAL MANIPULATION.

BY DR. A. DOUD, OF OLATHE, KAN.

At the present time the profession is urged to a higher standard of dental education, and the attention almost wholly directed to the discussion of educational and scientific problems. While a knowledge of anatomy, physiology and pathology is necessary in the preservation of the dental organs, it must be apparent that manipulative skill is equally important, and should not be slighted. There is but little gained by a correct diagnosis if one can not correct the difficulties, and but little credit to the doctorate to pass an examination on pathology, diagnosis, prognosis and therapeutics, if he does not understand the laws of mechanics and can not make a neat and permanent filling. A dentist should be more than a mechanic, and he must be more than a doctor. His office reputation and the value of his services depend more on his manipulative skill than on his knowledge of scientific subjects.

It is desirable to possess the ability to extract a tooth skillfully, without break or mishap of any kind, but a higher skill and one more difficult to attain, is that which protects those valuable organs from the forceps and saves them for future service. This skill is worthy of the highest ambition of every dentist, and whatever contributes to it ought on no account to be neglected.

In our observations of different operators we notice that they vary in their manner of performing the same work. There is a difference in the position at the chair, and in the manner of handling the instruments. With some the difference is very slight, and may denote different degrees of skill, but not always, for each one assumes that position which is most convenient and effective in directing the instrument. Some are more graceful than others perhaps, but each may reach the same results as readily and perfectly. But between the skillful manipulator and the bungler, there is so great a difference that no one can fail to distinguish one from the other. The one is easy and graceful and performs the most difficult operations promptly and thoroughly. He makes no false strokes and no mistakes. Each part of the work is executed in harmony with that which preceded it. The other shows his awkwardness in every motion, and finds the most simple operations difficult. His efforts are made hesitatingly and the results are usually unsatisfactory. His fingers refuse to perform the work assigned them, because they were never intended to be employed in that direction.

What is the cause of this decided difference? There are various reasons, but the principal one, we believe, is want of natural ability, and no amount of training can supply this deficiency. Some individuals are qualified only for the most rude and simple occupations, and when they move out of their natural range they become failures from beginning to end. A few of this class, by improperly estimating their abilities, find their way into the dental profession and not only do injustice to themselves and to their patients, but to the whole profession.

The dental profession requires fine artistic skill and delicate manipulations, and the dentist to be successful must possess good natural qualifications, though his final success depends on his acquired abilities. While we believe there are some who can not become skillful operators, yet a person of moderate ability, and even those who are considered awkward by their preceptors, can by patient perseverance and a stubborn determination to succeed, reach a high degree of excellence. Ambition, patience and perseverance are great elements of success. A lazy, slovenly person will not make a good dentist.

Mere manual dexterity must not be mistaken for professional skill. Some persons become skillful in a certain class of operations, by simply doing the same thing over again and again. As long as they can classify cases and reduce them to a certain fixed standard, they get along smoothly. But when a case presents so many variations as to require a different mode of treatment, they are lost, and are likely to fail. This class would make better carpenters than dentists, for then it would be proper for them to work by rule and model. Professional skill accepts no fixed rules, nor does it endeavor to modify cases in order to proceed upon some previously studied form of operation. It first seeks to understand the case, then devises the remedies. To do this readily, quickness of perception is necessary, that the operator may see at a glance the condition and know what to do. Whereas, if his ideas come slowly, his first operations will be done without knowing what he wants to do or how to do it.

Order in the arrangement of a dental office is essential. "A place for everything and everything in its place," so that the operator can lay his hand on the proper instrument without loss of time. To avoid confusion, only such instruments as are necessary for the operation should be allowed on the instrument stand. There need never be more than a half dozen, even for the most difficult case.

